# Hippocampal MicroRNAs Respond to Administration of Antidepressant Fluoxetine in Adult Mice

**DOI:** 10.3390/ijms19030671

**Published:** 2018-02-27

**Authors:** Nan Miao, Junghee Jin, Seung-Nam Kim, Tao Sun

**Affiliations:** 1 Center for Precision Medicine, School of Medicine and School of Biomedical Sciences, Huaqiao University, 668 Jimei Road, Xiamen 361021, China; miaonan@hqu.edu.cn; 2 Department of Cell and Developmental Biology, Cornell University Weill Medical College, 1300 York Avenue, Box 60, New York, NY 10065, USA; jjin@rockefeller.edu (J.J.); snkim@dongguk.edu (S.-N.K.); 3 College of Korean Medicine, Dongguk University, Ilsandonggu, Goyangsi 10326, Gyeonggido, Korea

**Keywords:** miRNAs, fluoxetine, antidepressant, depression, hippocampus

## Abstract

Current antidepressant treatments to anxiety and depression remain inadequate, burdened by a significant percentage of misuse and drug side-effects, due to unclear mechanisms of actions of antidepressants. To better understand the regulatory roles of antidepressant fluoxetine-related drug reactions, we here investigate changes of expression levels of hippocampal microRNAs (miRNAs) after administration of fluoxetine in normal adult mice. We find that 64 miRNAs showed significant changes between fluoxetine treatment and control groups by analyzing 626 mouse miRNAs. Many miRNAs in response to fluoxetine are involved in neural-related signaling pathways by analyzing miRNA-target gene pairs using the Kyoto encyclopedia of genes and genomes (KEGG) and Gene Ontology (GO). Moreover, miRNAs with altered expression are mainly associated with the repression of the dopaminergic synapse signals, which may affect hippocampal function after fluoxetine treatment. Our results demonstrate that a number of miRNAs respond to antidepressants even in normal mice and may affect target gene expression, which supports the safety consideration of inappropriate treatment and off-label use of antidepressant drugs.

## 1. Introduction

Depression is a major mood disorder characterized by chronic low mood, disturbance of sleep and appetite, as well as feelings of inferiority, despair, and suicide [1,2,3]. Recent studies on the mechanisms of depression are mainly concentrated on the genetic, neural and biochemical factors, neuroendocrine function, electroencephalogram (EEG) dynamics, neuroimaging and psychosocial problems [4]. Despite unremitting efforts of a few decades, the molecular and cellular mechanisms associated with depression remain unclear. No objective biomarkers are available for accurate evaluation in the therapeutic effect of antidepressant treatment.

Antidepressant drugs, including monoamine oxidase inhibitors (MAOIs) and tricyclic antidepressants (TCAs), were first used in the 1950s. Fluvoxamine was launched in the European market in 1983 and in the US market in 1994 [5]. However, due to their nonspecific interactions with multiple receptors in the central nervous system (CNS), they exhibit unwanted side effects that limit their use in the clinics [6,7,8,9]. Selective serotonin reuptake inhibitors (SSRIs), the well-developed third-generation antidepressants, have been proved to be more effective [10,11,12]. Among them, fluoxetine has the longest half-life and well absorption after oral administration [12,13,14,15]. Since then, antidepressants have been increasingly used, more than two dozen antidepressants have been approved to treat mood and anxiety disorders, and one in 10 people takes them [16,17]. Side effects of antidepressants are usually underreported in clinical trials, and a small but growing literature on the inappropriate use of antidepressants largely consists of case reports. Furthermore, individuals without professional diagnosis may be vulnerable to misuse or abuse of medications [18].

MicroRNAs (miRNAs) are a family of noncoding RNA molecules, and regulate different biological pathways by negatively modulating target gene expression [19]. Since one miRNA may regulate up to hundreds of genes, and collectively 50–60% of total transcriptomes, miRNAs could have pleiotropic biological effects [20,21]. In mammals, miRNAs have been shown as one of the key regulators that control development of the CNS, including neurogenesis, neuronal proliferation and synaptic plasticity [22,23,24,25]. Disruptions to these processes have been linked to development of depression [26]. The involvement of miRNAs in the response to fluoxetine is only beginning to be explored, for example, miR-16 mediated the action of fluoxetine by acting as a micromanager of hippocampal neurogenesis [27,28,29]. The therapeutic action of fluoxetine in shocked mice was associated with a significant reduction of miR-1971 in the cortex [30]. Moreover, studies have shown that miRNAs may function as pre-diagnosis markers in different diseases via post-transcriptional regulation, such as cardiovascular disorders, meiosis-associated cancer and reproductive dysfunction [31,32,33,34,35].

In this study, we show that 39 miRNAs are up-regulated (signal > 500) and 25 miRNAs are down-regulated (signal > 500) after the antidepressant fluoxetine administration of normal mice by using an Affymetrix microarray analysis. We also demonstrate potential roles of up- and down-regulated miRNAs in hippocampus by analyzing miRNA predicted targets using Kyoto encyclopedia of genes and genomes (KEGG) and Gene Ontology (GO). Finally, we find that changes of miRNAs are associated with the repression of the dopaminergic synapse signal, which could cause fluoxetine-related dopaminergic abnormal inhibition.

## 2. Results

### 2.1. Differential Expression of miRNAs upon Fluoxetine Treatment in Adult Mice

Our previous study has shown that miRNAs are associated with anxiety- and depression-like behaviors in mice [36]. To test whether antidepressant itself has an effect on expression of miRNAs in hippocampus in wild type mice, adult C57BL/6 mice were directly administrated daily fluoxetine or vehicle for 2 weeks. Hippocampus was dissected for extracting RNA and then miRNA microarray analyses.

**Figure 1:**
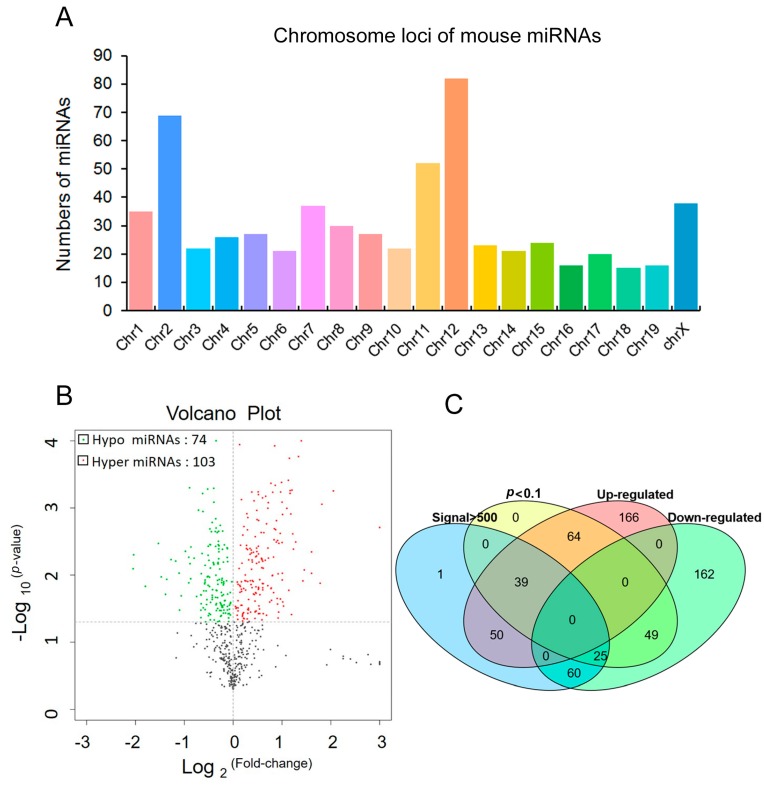
MiRNA expression in hippocampi of antidepressant administration and control mice. (**A**) The numbers of miRNAs in mouse chromosome loci in Affymetrix array analysis; (**B**) Volcano plot of miRNA expression levels in antidepressant administration and control group. Each point represents a miRNA, “red” dot means hyper, “green” dot means hypo expression and “black” dot means none significance (antidepressant vs. control); (**C**) Venn diagram of miRNA array results. The ellipses in different color (blue, yellow, red and green) represent miRNAs in four groups: signal > 500 (175 miRNAs), *p* < 0.1 (177 miRNAs), up-regulated (319 miRNAs) and down-regulated (296 miRNAs), respectively. The overlapping parts of ellipses represent the intersection of different groups, such as 103 and 74 miRNAs were in up-regulated and down-regulated groups (*p* < 0.1), respectively.

**Figure 2:**
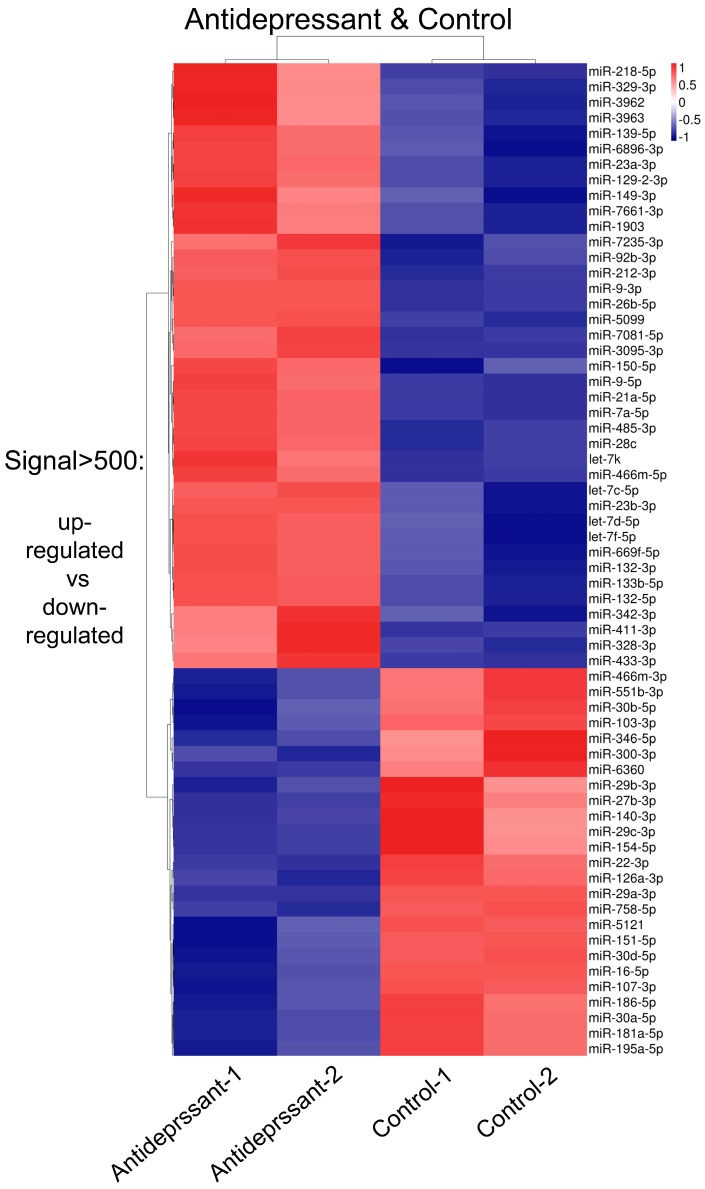
The heatmap of normalized expression of miRNAs in hippocampi of antidepressant administration and control mice to distinguish the difference of expression. The heatmap was drawn with normalized expression ((x − min)/(max − min)) of each miRNA. The “x” means the expression of each miRNA, “min” means the lowest expressed miRNA and “max” means the highest expressed miRNA. Blue, white and red indicate relatively low, middle and high expression of miRNAs, respectively.

1892 miRNA probes were tested in the microarray analysis (Tables S1 and S2). 623 known mature miRNAs have been mapped randomly in the mouse chromosomes through the mouse miRBase (Figure 1A and Figure S1, and Table S3). To screen differentially expressed miRNAs, the transcriptional level of miRNAs was compared between hippocampi of the antidepressant treated and control groups (Figure 1B and Table S3). Altogether, while 103 and 74 miRNAs were highly expressed in the antidepressant treated and control groups (*p* < 0.1), respectively, 446 of 623 miRNAs did not show differential expression (Figure 1B and Table S3). When the signal of differentially expressed miRNAs was set larger than 500 and the two fold-change cutoff was adjusted *p*-value as *p* < 0.1, 64 miRNAs (39 up-regulated miRNAs and 25 down-regulated miRNAs) were found to show differential expression in the antidepressant treated and control groups (Figure 1C and Figure 2, and Table 1).

Among 64 miRNAs (signal > 500, *p* < 0.1), 39 miRNAs exhibited higher expression levels in the antidepressant treated group than the control (termed up-regulated miRNA group or hyper miRNA group) (Figure 1B,C and Figure 2, Table 1). The well-studied miRNAs within this group included let-7 family (let-7c/d/f/k), miR-212 cluster (miR-212-3p and miR-132-3p/5p), miR-23a/b, miR-9-3p/5p, miR-411 clusters (miR-299a and miR-329) and miR-466 clusters (miR-466m-5p and miR-669f-5p) (Figure 2 and Table 1). On the other hand, 25 miRNAs were expressed at a lower level in the antidepressant treated group than the control (termed down-regulated miRNA group or hypo miRNA group). This group included miR-30 family (miR-30a/b/d) and miR-29 family (miR-29a/b/c). Overall, identification of 64 miRNAs with significant changes after the fluoxetine administration in normal adult mice suggests that a considerable number of miRNAs respond to antidepressants in hippocampus.

### 2.2. Prediction of miRNA Target Genes and Pathway Analysis

Because miRNAs normally function through silencing their target genes, we next searched targets of differentially expressed miRNAs. Using TargetScan (http://www.targetscan.org/vert_71/) and miRDB (http://www.mirdb.org/), 7478 genes were predicted as targets of the 39 up-regulated miRNAs, and 3404 genes as targets of 25 down-regulated miRNAs (Table S4).

**Figure 3:**
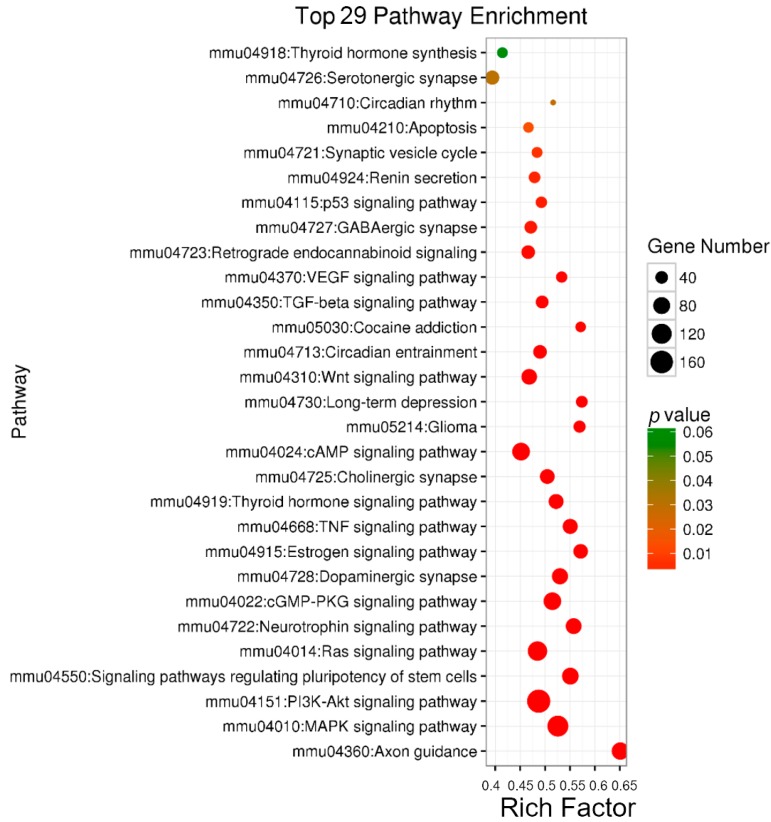
The bubble chart of up-regulated miRNAs in KEGG analysis. *Y*-axis represents the pathway name, *X*-axis represents the rich factor, the size of bubble represents the number of genes, and the color of bubble represents the *p*-value. VEGF: vascular endothelial growth factor; TGF: transforming growth factor; Wnt: wingless-type MMTV integration site; cAMP: cathelicidin antimicrobial peptide; TNF: tumor necrosis factor; cGMP-PKG: cyclic GDP-mannose pyrophosphorylase dependent protein kinase; Ras: rat sarcoma viral oncogene homolog; PI3K: phosphatidylinositol 3-kinase; Akt: RAC serine/threonine-protein kinase; MAPK: mitogen-activated protein kinase.

To comprehensively study the interaction between miRNAs and their predicted targets, analyses of KEGG pathway for the miRNA-target pairs were performed in miRNAs of up-regulated and down-regulated groups (Tables S5 and S6). We found that some enriched pathways are associated with neural development and function, for example pathways in the endocrine system development and neurotrophin signaling (Figure 3 and Figure S2). Pathways related to hippocampal neurogenesis for the predicted targets of up-regulated miRNAs included genes involved in axon guidance, dopaminergic synapse, long-term depression and GABAergic synapse (Figure 3). Predicted targets for the down-regulated miRNAs were associated with the wingless-type MMTV integration site (Wnt) signaling pathway and cholinergic synapse (Figure S2). These results suggest that many miRNAs in response to fluoxetine may participate in neural-related signaling pathways.

### 2.3. GO Analysis of Predicted Targets for miRNAs

**Figure 4:**
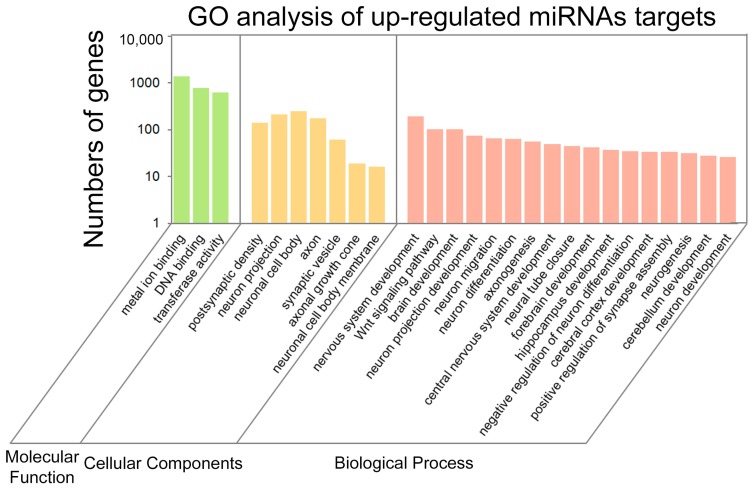
The histogram of target genes for up-regulated miRNAs in GO analysis.

To make a better understanding of biological processes of predicted targets for miRNAs, Gene Ontology (GO) analyses were performed in up-regulated and down-regulated miRNA targets that are associated with biological process (BP), cellular components (CC) and molecular function (MF). We found that 923 GO items (567 BP items, 155 CC items and 201 MF items) were enriched in up-regulated miRNAs (Tables S7–S9), and 614 GO items (395 BP items, 114 CC items and 105 MF items) were abundant in down-regulated miRNAs (Tables S10–S12). Among GO items that are associated with neurogenesis (Tables S13 and S14), some items of BP in up-regulated miRNAs were involved in functions regulating postsynaptic density, neuronal projection and synaptic vesicle, and some items of CC were involved in regulating neuronal projection, migration and differentiation (Figure 4). Moreover, the most significant GO terms in down-regulated miRNAs were related to synapse, axon and synaptic membrane (Figure S3). These results indicate that many up and down-regulated miRNAs are associated with hippocampal neurogenesis.

### 2.4. Functional Comparison of Predicted Targets for Up-Regulated and Down-Regulated miRNAs

**Figure 5:**
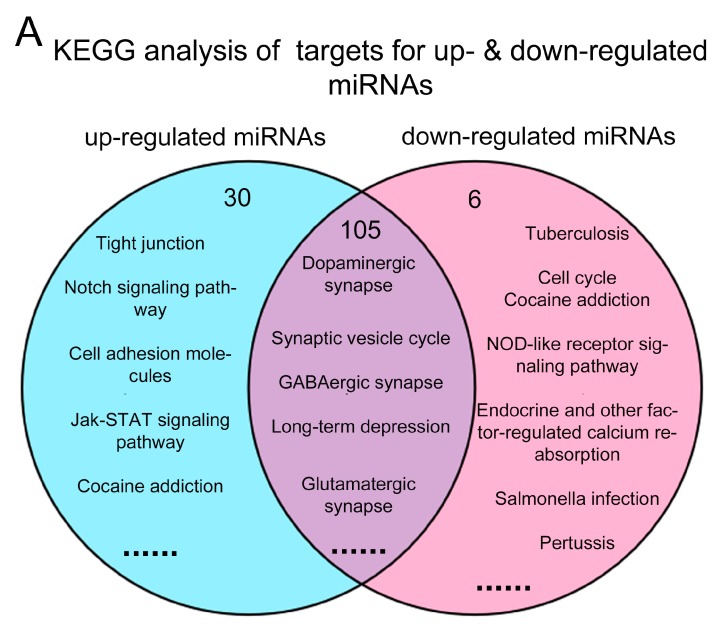

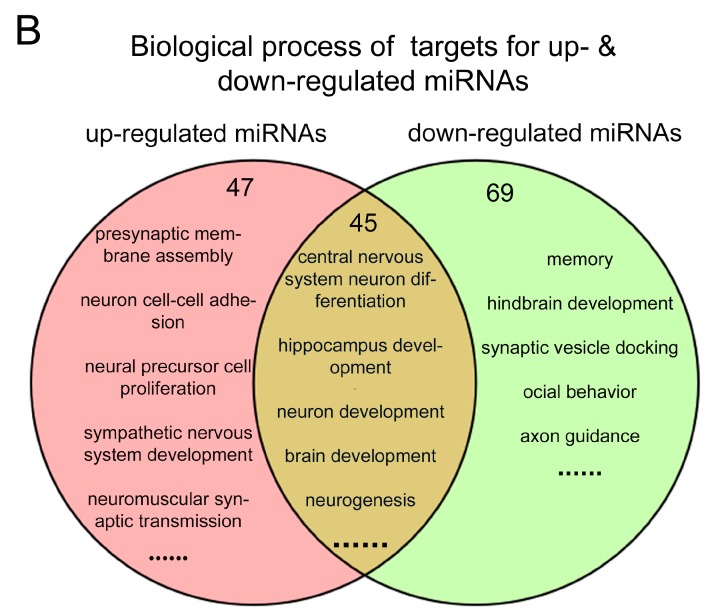
Venn diagrams of the comparison analysis of target genes for up- and down-regulated miRNAs. (**A**) Venn picture of GO analysis; (**B**) Venn picture of KEGG analysis. Jak-STAT: Janus kinase/signal transducers and activators of transcription; NOD: nucleotide binding oligomerization domain.

To study functional correlations of predicted targets between up-regulated and down-regulated miRNAs, we validated the overlapping KEGG and GO items among these targets (Figure 5).

In the KEGG analysis, most of the pathways were overlapped between two groups (105 out of 135 in up-regulated miRNAs and 105 out of 111 in down-regulated miRNAs) (Figure 5A and Table S16). Many of them were involved in neural development, for instance the dopaminergic synapse, synaptic vesicle cycle, GABAergic synapse and long-term depression. 30 pathways were specific abundant in up-regulated miRNAs, for example the tight junction, notch signaling pathway and cell adhesion molecules, while only 6 pathways were enriched in down-regulated miRNAs.

In the GO analyses of BP, 92 items in up-regulated miRNAs and 114 items in down-regulated miRNAs were found to be associated with neural development (Figure 5B, Tables S13–S15). 47 and 69 items were specifically abundant in up-regulated and down-regulated miRNAs, respectively. 45 GO items were found overlapping in up- and down-regulated miRNAs, for example neuronal differentiation and hippocampal development.

These functional analyses indicate that most GO and KEGG are overlapping in the predicted targets of up-regulated and down-regulated miRNAs.

### 2.5. miRNA Interaction Network for Elements Involved in Dopaminergic Synapse Signals

**Figure 6:**
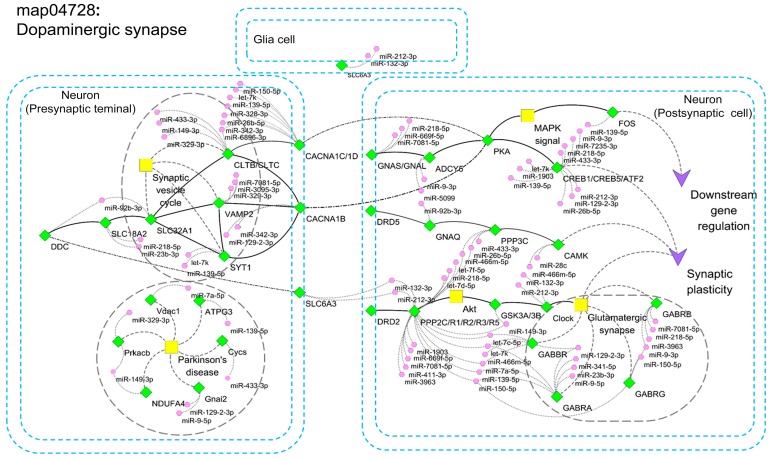
Network of miRNA-target pathways involved in dopaminergic synapse for up-regulated miRNAs. The rhombus represents the genes, hexagon represents the miRNAs and the square represents the main pathways in dopaminergic synapse. The solid lines with arrows represent the relationship between genes and pathways, and the dot lines represent the relationship between genes and miRNAs. The solid lines without arrows represent the sub-pathways. The double blue solid line represent the cell structure of neuron (presynaptic terminal and postsynaptic cell) and glia cell.

Previous studies have shown that antidepressant targeted Na^+^/Cl^−^-coupled dopaminergic neurotransmitter uptake defines a key therapeutic strategy to treat clinical depression and neuropathic pain [37,38,39,40]. We found that 71 predicted targets of up-regulated miRNAs and 39 predicted targets of down-regulated miRNAs are involved in the dopaminergic synapse signals (Figure 3 and Figure S4, and Table S17). Overall, 26 predicted targets were overlapped in these two groups, 45 and 13 predicted targets were specifically abundant in up-regulated and down-regulated miRNAs that are involved in regulating dopaminergic synapse, respectively (Figure S4 and Table S17). Interestingly, we found that many miRNAs (18 of 21) negatively regulate dopaminergic synapse pathways by inhibiting the positive genes (Figure 6). Moreover, some sub-pathways in hippocampal neurons might be modulated by up-regulated miRNAs, such as synaptic vesicle cycle, glutamatergic synapse, mitogen-activated protein kinase (MAPK) signal, and RAC serine/threonine-protein kinase (Akt) signal. Some miRNAs may alter the positive regulators that are associated with Parkinson’s disease.

These results suggest that miRNAs affected by the antidepressant fluoxetine in the adult hippocampus largely regulate target genes controlling dopaminergic synapse.

## 3. Discussion

Current antidepressant treatments remain inadequate, burdened by a significant percentage of misuse and side-effects. The fluoxetine may be deleterious to immature or undifferentiated neural cells, but the mechanisms are unclear [41,42,43]. Our study shows the diverse changes of miRNAs in hippocampus after the fluoxetine administration of normal mice. Some up-regulated miRNAs may negatively regulate the dopaminergic synapse pathway. Our results suggest that miRNAs respond to antidepressants in hippocampus of normal mice by inhibiting a wide range of target genes involved in many aspects of neural development and functions.

Studies have shown that miRNA is one of the most important prognosis markers in different clinical scenario. miR-181a-5p/miR-21a-5p ratios were used to detect mycotrophic lateral sclerosis with 90 and 85% sensitivity in cerebrospinal fluid [44]. miRNAs have been found to be involved in cardiac resynchronization therapy through association with cardiac angiogenesis (miR-30, miR-92 and miR-145), cardiac apoptosis (miR-30) and cardiac fibrosis (miR-29) [35]. Recently, several studies have reported that dysregulation of miRNAs may precipitate or aggravate anxiety and depression. For example, miR-17-92 knockout mice show anxiety- and depression-like behaviors, whereas miR-17-92 overexpressing mice exhibit anxiolytic and antidepressant-like behaviors [36]. Higher or lower levels of miR-135 can alter anxiety- and depression-like behaviors in mice via regulating activity of serotonergic (5HT) neurons, 5HT levels in blood and brain, and behavioral response to antidepressant treatment [45]. Fluoxetine is a widely used antidepressant in treatment of mood and anxiety disorders via regulating miR-29 family and genes such as brain derived neurotrophic factor (*BDNF*), response regulator in two-component regulatory system with CreC (*CREB*), and HUS-associated diffuse adherence (*HDACs*) [46,47,48,49]. However, the involvement of miRNAs in the response to antidepressants in adult hippocampus is only beginning to be explored. Previous studies have shown that miR-17-92 mediates antidepressant-regulated adult hippocampal neurogenesis, which is verified by quantitative reverse transcription PCR (qRT-PCR) and behavioral tests [36]. We identified 64 miRNAs with significant changes after the fluoxetine administration in normal adult mice, suggesting that a large number of miRNAs respond to antidepressants in hippocampus. Even though expression of some of these miRNAs have been validated by qRT-PCR, a thorough verification will draw a comprehensive profile of miRNA expression in the future.

Among altered miRNAs, some of them have been shown to play roles in brain functions. For example, the fluoxetine induced up-regulation of let-7 family (let-7c/d/f/k) and miR-23a/b have been shown to have high risk of temporal lobe epilepsy [50]. Abnormal ascending of miR-132/212 alters memory and learning by modulating dendritic growth and arborization of newborn neurons via attenuating hippocampal transcriptome [51,52,53]. In addition, reduced expression of miR-29 family causes several neurodegenerative disorders, including Alzheimer’s disease, Huntington’s disease, and spinocerebellar ataxias, by targeting voltage-dependent anion channel 1 (*VDAC1*), β-secretase 1 (*BACE1*) and neuron navigator 3 (*NAV3*) [54,55,56,57]. Interestingly, these miRNAs were also found up- or down-regulated after fluoxetine treatment in the hippocampus.

Since miRNAs function through silencing target genes, KEGG and GO analyses of targets for altered miRNAs after fluoxetine treatment, have identified some pathways important for neural development and function. We have found that miR-16 is down-regulated, and its predicted targets, including *BDNF*, apoptosis regulator Bcl-2 (*BCL-2*), Serotonin transporter (*SERT*), and Wnt2, have been shown to regulate hippocampal response to serotonin reuptake inhibitor (SRI) antidepressants [27,58,59,60]. Even though the functional relationship between altered miRNAs and their targets needs to be further verified, the KEGG and GO analyses have provided a reference for future identification of miRNA-target interactions responding to antidepressants. Because the false positive of miRNA target prediction is inevitable, the future work will be to validate miRNA-target regulation and to examine the biological meaning of these interactions.

Moreover, we have found that many pathways of predicted targets are overlapped between up- and down-regulated miRNAs, which suggests a counter-balanced functional output. Interestingly, the dopaminergic synapse signal appears to be suppressed in hippocampal neurons by miRNAs after fluoxetine treatment, suggesting that fluoxetine may be deleterious to normal dopaminergic neurons by abnormal inhibition [61,62]. Some miRNA-target pairs in dopaminergic synapse signal have been reported. For example, miR-9-*CREB* negative feedback minicircuitry plays a critical role in the determination of proliferation and migration in glioma cells [63,64]; miR-433-3p suppresses cell growth and enhances chemosensitivity by targeting *CREB* in human glioma [65]; miR-26a/Kruppel like factor 4 (*KLF4*) and cAMP responsive element binding protein CCAAT/enhancer binding protein (*CREB-C/EBPβ*) signaling pathways regulate survival of mycobacterium tuberculosis in macrophages [66]. Since CRE-mediated transcription promotes neuronal plasticity, some up-regulated miRNAs (miR-9, miR-433-3p and miR-26a) may play essential roles in fluoxetine-induced dopaminergic synapse suppression by targeting *CREB* [67,68,69]. We have found that fluoxetine treatment in hippocampus of wild type mice causes up-regulation of miRNAs that normally suppress the dopaminergic synapse signal, which might in turn regulate hippocampal function related to anxiety and depression.

In summary, this study has identified many up- and down-regulated hippocampal miRNAs after the fluoxetine administration in wild type mice, suggesting that a large number of miRNAs respond to antidepressants even under normal conditions. Subsequently, a broad range of target genes are affected by altered miRNAs. This data further suggests safety considerations related to antidepressant usage. Further analyses of these altered miRNAs and related pathways identified in this study will help to promote the understanding of molecular mechanisms involved in inappropriate use of antidepressants in the future.

## 4. Materials and Methods

### 4.1. Antidepressant Treatment

The C57BL/6 mice from the same parents was fed in the same condition (temperature, humidity, fodder and etc.). The adult mice were administrated daily fluoxetine (20 mg/kg, Sigma-Aldrich, St. Louis, MO, USA) or vehicle for 2 weeks (13-week-old) until the day before the experiment [36]. Fluoxetine was dissolved in dimethylsulfoxide (DMSO) and then diluted in saline. The drug was finally diluted in 100 μL of 0.9% saline for administration [36,70]. Animal use was overseen by the Animal Facility at Weill Cornell Medical College (#2011-0062, 25 July 2011), and was performed according to the institutional ethical guidelines for animal experiments.

### 4.2. RNA Preparation

The adult hippocampi of the 13-week-old mice were dissected from control group and fluoxetine administration group, each of them from a different litter. To avoid contamination of cortical tissues, the adult hippocampi including the Cornu Ammonis 1 (CA1), CA2, CA3, CA4 areas and the dentate gyrus (DG) were collected through fine dissection from the cerebral cortex. All tissue samples were washed briefly with phosphate buffered solution (PBS), and total RNA from each brain was isolated by using Trizol reagent (Invitrogen, Carlsbad, CA, USA). The quality and quantity of these RNA samples were determined by NanoDrop spectrophotometer (ND-2000C, Thermo Fisher Scientific Inc., Wilmington, DE, USA). Agilent RNA 6000 Nano assay (Agilent Technologies, Inc., Santa Clara, CA, USA) and agarose gel electrophoresis were used to check purity of the RNA samples.

### 4.3. miRNA Microarray Assay

A commercial Affymetrix array service (LC Sciences, LLC, Houston, TX, USA) was used for miRNA array analyses. The labeled RNA was hybridized to the Affymetrix GeneChip miRNA1.0 array (Affymetrix, Santa Clara, CA, USA) containing 1917 miRNA probes, and the array was later scanned by Axon 4000B (Axon Instruments, Union City, CA, USA) from Molecular Devices (San Jose, CA, USA). The RNA labeling, miRNA array hybridization, and quantification were performed via Affymetrix GeneChip system instruments and protocols. The sequence for the array result was selected from the miRBase database 21 (http://www.mirbase.org).

### 4.4. Microarray Data Analysis

The data analysis of miRNAs expression profiling (data filtering, normalization and statistical calculations) was processed by R (R-Foundation for Statistical Computing, Vienna, Austria; version 2.12.1). Between 2 antidepressant treatments and 2 control tissues, the significant difference of miRNAs expression was selected by fold-change and a *p*-value with the following criteria: fold-change > 1 and *p*-value < 0.1 [71]. The heatmap was performed with normalized expression ((x − min)/(max − min)) of each miRNA by pheatmap package (https://cran.r-project.org/web/packages/pheatmap/index.html) of R language. The heat map was drawn. Blue, white and red indicate relatively low, middle and high expression of miRNAs, respectively.

### 4.5. miRNA Target Prediction

Target prediction of miRNAs was performed using a combination of 2 prediction programs including miRDB and TargetScan [72]. The significantly changed miRNAs were analyzed with their chromosome location and *p*-value using miRBase [73].

### 4.6. miRNA Target Functional Analysis

Predicted target genes were imported into the Database for Annotation, Visualization and Integrated Discovery (DAVID) v6.8 (https://david.ncifcrf.gov/), an online functional annotation tools for investigators to understand biological meaning. We performed Gene Ontology (GO) and KEGG analysis of the miRNAs-targets with differential expression. In this study, modules in the gene-miRNA-KEGG were mined with Cytoscape 3.6 (http://www.cytoscape.org/) and then functional enrichment analysis was applied to the miRNAs in the modules.

### 4.7. Statistical Analysis

The *p*-value to verify significant difference is set to <0.1 in array result. *p*-values were determined by unpaired Student’s *t*-test for assessing the significance of differences between two treatments. *p*-values < 0.05 were considered significant.

## Figures and Tables

**Table 1:** Up-regulated and down-regulated hippocampal miRNAs upon fluoxetine treatment.

**Up-Regulated miRNAs**			
**miRNA Name (Fold-Change, Fold-Change = Treatment/Control)**			
miR-7081-5p (22.83)	miR-5099 (1.71)	miR-485-3p (1.40)	miR-3095-3p (1.23)
miR-3962 (3.43)	let-7k (1.64)	let-7f-5p (1.39)	let-7c-5p (1.23)
miR-133b-5p (2.27)	miR-92b-3p (1.61)	let-7d-5p (1.39)	miR-466m-5p (1.22)
miR-21a-5p (2.14)	miR-1903 (1.53)	miR-411-3p (1.39)	miR-23b-3p (1.20)
miR-3963 (2.01)	miR-150-5p (1.50)	miR-23a-3p (1.37)	miR-149-3p (1.20)
miR-26b-5p (1.80)	miR-328-3p (1.50)	miR-7a-5p (1.34)	miR-342-3p (1.19)
miR-132-3p (1.79)	miR-6896-3p (1.49)	miR-7235-3p (1.30)	miR-9-5p (1.16)
miR-212-3p (1.75)	miR-132-5p (1.44)	miR-433-3p (1.30)	miR-341-5p (1.15)
miR-329-3p (1.75)	miR-129-2-3p (1.43)	miR-139-5p (1.27)	miR-7661-3p (1.13)
miR-218-5p (1.74)	miR-28c (1.40)	miR-669f-5p (1.27)	
**Down-Regulated miRNAs**			
**miRNA Name (Fold-Change, Fold-Change = Control/Treatment)**			
miR-466m-3p (1.09)	miR-22-3p (1.23)	miR-30d-5p (1.39)	miR-103-3p (1.22)
miR-151-5p (1.09)	miR-107-3p (1.23)	miR-195a-5p (1.42)	miR-30b-5p (1.32)
miR-300-3p(1.1)	miR-27b-3p (1.24)	miR-5121 (1.43)	miR-186-5p (1.55)
miR-126a-3p (1.16)	miR-346-5p (1.24)	miR-758-5p (1.44)	miR-551b-3p (1.23)
miR-140-3p (1.18)	miR-16-5p (1.25)	miR-29c-3p (1.50)	
miR-154-5p (1.22)	miR-29a-3p (1.27)	miR-29b-3p (1.50)	
miR-181a-5p (1.22)	miR-30a-5p (1.38)	miR-6360 (2.26)	

## Supplementary Materials

Supplementary materials can be found at http://www.mdpi.com/1422-0067/19/3/671/s1.
