# Supplementary material for: Hippocampal MicroRNAs Respond to Administration of Antidepressant Fluoxetine in Adult Mice
Source: Int J Mol Sci. 2018 Feb 27;19(3):671. doi: 10.3390/ijms19030671 (PMC5877532; doi:10.3390/ijms19030671)
Supplement: Supplementary file 1 [file ijms-19-00671-s001.zip › ijms-271078-supplementary materials/ijms-271078-Supplementary Materials.docx]

Supplementary Materials: Hippocampal MicroRNAs Respond to Administration of Antidepressant Fluoxetine in Adult Mice

Nan Miao^1^, Junghee Jin^2^, Seung-Nam Kim^2^, and Tao Sun^1,2,*^

^1^ Center for Precision Medicine, School of Medicine and School of Biomedical Sciences, Huaqiao University, 668 Jimei Road, Xiamen, Fujian 361021 China.

^2^ Department of Cell and Developmental Biology, Cornell University Weill Medical College, 1300 York Avenue, Box 60, New York, NY 10065 USA

* Corresponding author: Dr. Tao Sun, Email: taosun@hqu.edu.cn.

**Supplementary Tables**

**Table.S1** S130251_MultiArray Analysis Data

**Table.S2** MRA-1002_miRMouse_20

**Table.S3** T-test of antidepressant and control group

**Table.S4** Target prediction of up-regulation & down-regulation miRNAs

**Table.S5** The KEGG analysis of down-regulated miRNAs targets

**Table.S6** The KEGG analysis of up-regulated miRNAs targets

**Table.S7** The GO analysis of up-regulated miRNAs in biological process (BP)

**Table.S8** The GO analysis of up-regulated miRNAs in cellular component (CC)

**Table.S9** The GO analysis of up-regulated miRNAs in molecular function (MF)

**Table.S10** The GO analysis of down-regulated miRNAs in biological process (BP)

**Table.S11** The GO analysis of up-regulated miRNAs in cellular component (CC)

**Table.S12** The GO analysis of up-regulated miRNAs in molecular function (MF)

**Table.S13** The GO items associated with neural system development in up-regulated miRNAs targets

**Table.S14** The GO items associated with neural system development in down-regulated miRNAs targets

**Table.S15** The Venn picture of comparison analysis of GO in down-regulated and up-regulated miRNAs targets

**Table.S16** The Venn picture of comparison analysis of KEGG in down-regulated and up-regulated miRNAs targets

**Table.S17** The Venn picture of putative targets in in Dopaminergic synapse of down-regulated and up-regulated miRNAs

**Supplementary Figures**


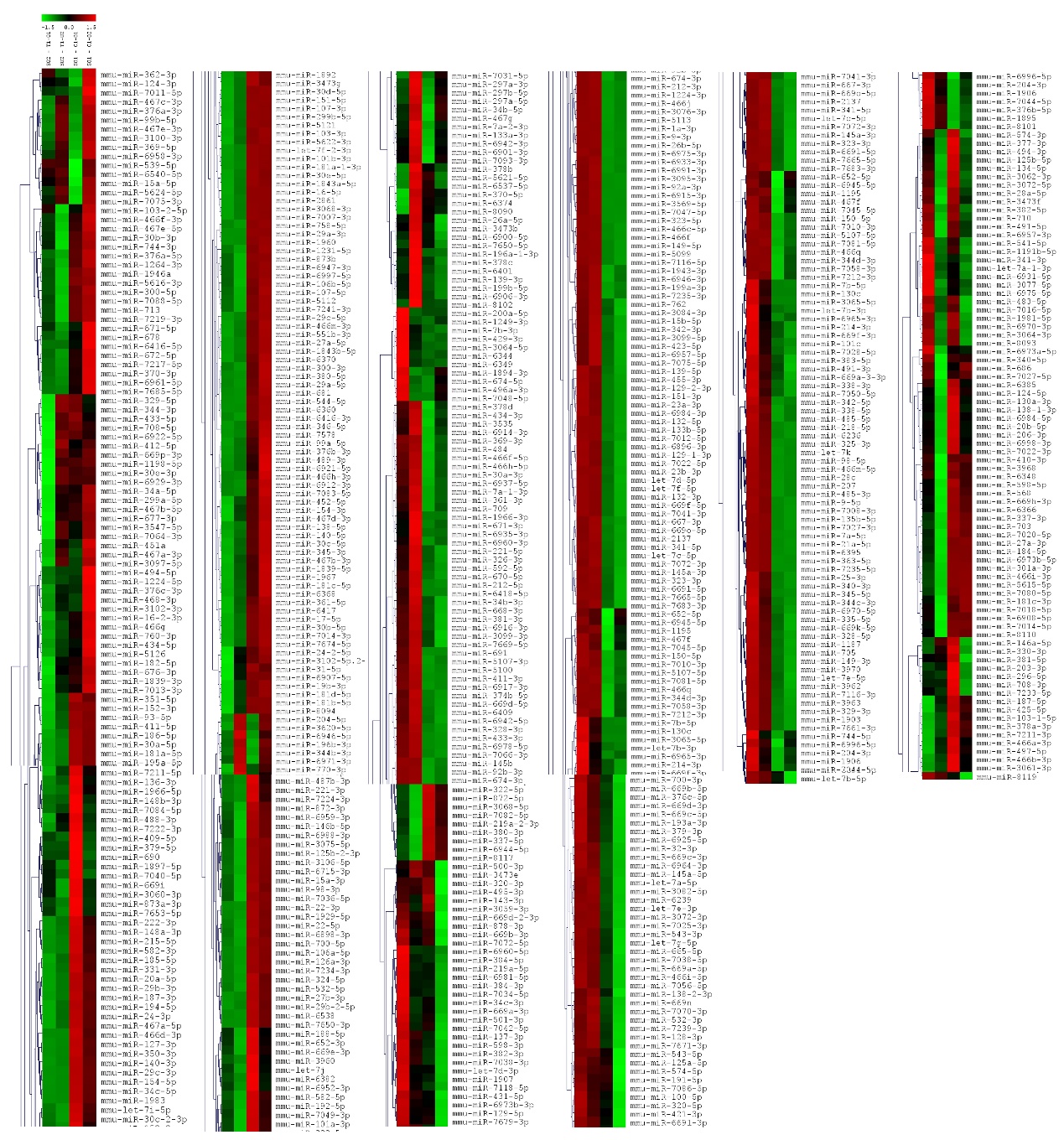


**Figure S1.** The heat maps of all miRNAs in the microarray analyses. The heat map was drawn with relative expression of each miRNAs. Green, black and red indicate low, middle and high expression level of miRNAs, respectively. Color map is used to distinguish the difference of expression.


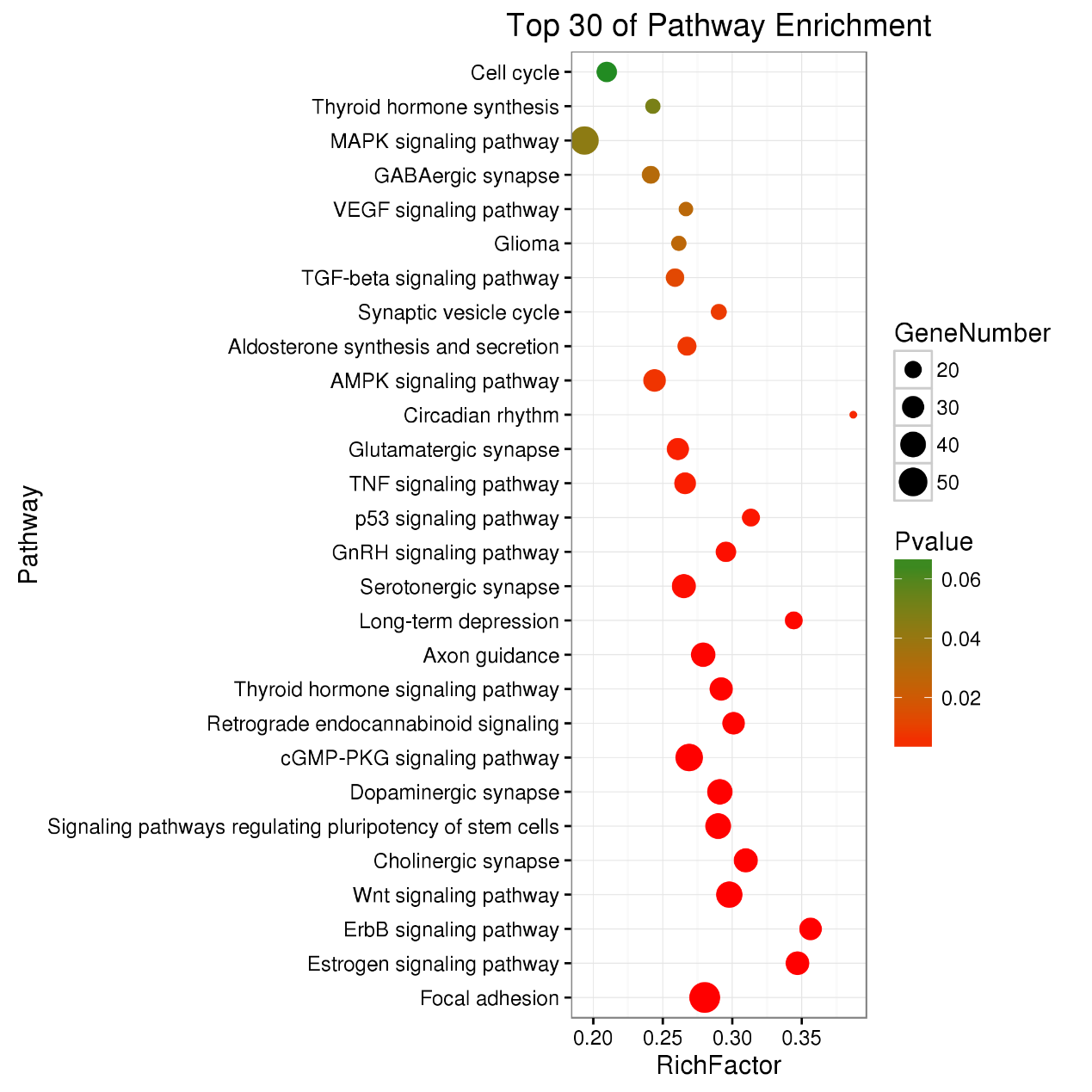


**Figure S2.** The bubble chart of down-regulated miRNAs in KEGG analysis. Y-axis represents the pathway name，X-axis represents the Rich factor, the size of bubble represents the number of genes, and the color of bubble represents the Q-value.


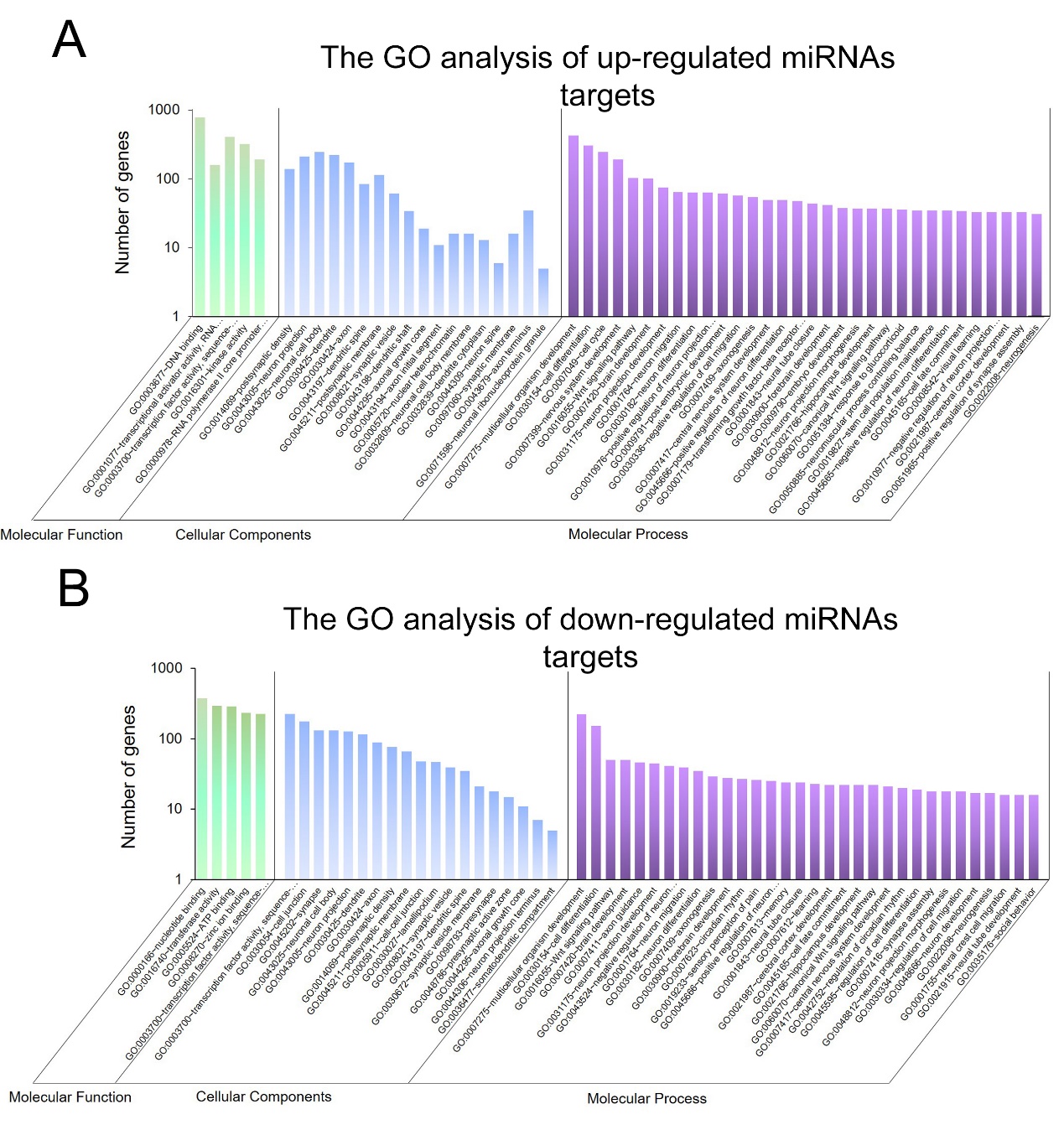


**Figure S3.** The histogram of up-regulated and down-regulated miRNAs in GO analysis. The GO analysis of up-regulated miRNAs. The GO analysis of down-regulated miRNAs.


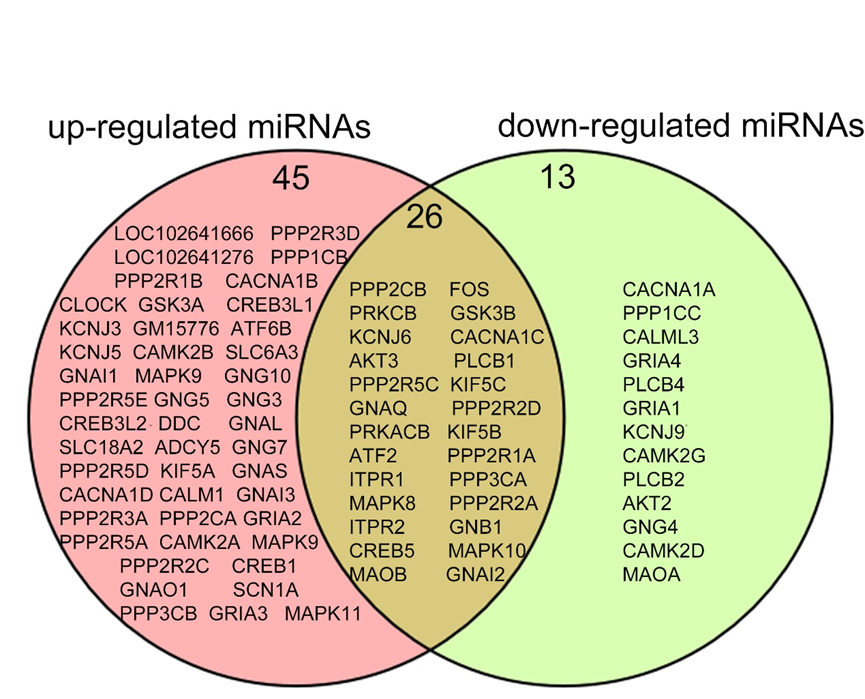


**Figure S4.** The Venn diagrams of predicted targets involved in dopaminergic synapse for up- and down-regulated miRNAs. Venn picture of miRNA array results. The ellipse in different colors represents targets for miRNAs in up-regulated and down-regulated groups.
